# Molecular Genetic Characterization of Patients With Focal Epilepsy Using a Customized Targeted Resequencing Gene Panel

**DOI:** 10.3389/fneur.2018.00515

**Published:** 2018-07-06

**Authors:** Meng-Han Tsai, Chung-Kin Chan, Ying-Chao Chang, Chih-Hsiang Lin, Chia-Wei Liou, Wen-Neng Chang, Ching-Ching Ng, Kheng-Seang Lim, Daw-Yang Hwang

**Affiliations:** ^1^Department of Neurology, Kaohsiung Chang Gung Memorial Hospital, College of Medicine, Chang Gung University, Kaohsiung, Taiwan; ^2^Genetics and Molecular Biology, Faculty of Science, Institute of Biological Sciences, University of Malaya, Kuala Lumpur, Malaysia; ^3^Department of Pediatrics, Kaohsiung Chang Gung Memorial Hospital, Kaohsiung, Taiwan; ^4^Division of Neurology, Faculty of Medicine, University of Malaya, Kuala Lumpur, Malaysia; ^5^Division of Nephrology, Kaohsiung Medical University Hospital, Kaohsiung Medical University, Kaohsiung, Taiwan

**Keywords:** focal epilepsy, multigene panel, targeted resequencing, NGS, multiplex PCR

## Abstract

**Objective:** Focal epilepsy is the most common subtype of epilepsies in which the influence of underlying genetic factors is emerging but remains largely uncharacterized. The purpose of this study is to determine the contribution of currently known disease-causing genes in a large cohort (*n* = 593) of common focal non-lesional epilepsy patients.

**Methods:** The customized focal epilepsy gene panel (21 genes) was based on multiplex polymerase chain reaction (PCR) and sequenced by Illumina MiSeq platform.

**Results:** Eleven variants (1.85%) were considered as pathogenic or likely pathogenic, including seven novel mutations. There were three *SCN1A* (p.Leu890Pro, p.Arg1636Ter, and p.Met1714Val), three *PRRT2* (two p.Arg217Profs^*^8 and p.Leu298Pro), two *CHRNA4* (p.Ser284Leu, p.Ile321Asn), one *DEPDC5* (p.Val516Ter)*, one PCDH19* (p.Asp233Asn), and one *SLC2A1* (p.Ser414Ter) variants. Additionally, 16 other rare variants were classified as unknown significance due to inconsistent phenotype or lack of segregation data.

**Conclusion:** Currently known focal epilepsy genes only explained a very small subset of focal epilepsy patients. This indicates that the underlying genetic architecture of focal epilepsies is very heterogeneous and more novel genes are likely to be discovered. Our study highlights the usefulness, challenges and limitations of using the multi-gene panel as a diagnostic test in routine clinical practice in patients with focal epilepsy.

## Introduction

Focal epilepsy constitutes for about 60% of all epilepsies, which is the commonest phenotypic group of epilepsies (1). The etiology of more than half of the focal epilepsies remains uncertain despite high-quality neuroimaging studies (2–4). Some of these unsolved focal epilepsy patients may have a genetic etiology. Recently, patients with focal structural epilepsies were also found to have a genetic cause, such as mTOR pathway genes mutations in focal cortical dysplasia (5, 6). Several disease-causing genes were identified in patients presented with focal seizures as part of their phenotypic spectrums through studies of large families (7–12). For examples, *LGI1* in familial lateral temporal epilepsies (13), *DEPDC5* in familial focal epilepsy with various foci (9, 10), *SCN1A* in genetic epilepsy with febrile seizure plus (GEFS+) (14, 15) and *CHRNA2, CHRNAB2, CHRNA4, KCNT1* in sleep related hypermotor epilepsies (16–19). A better understanding of the contribution of these genes in common focal epilepsies patients can be helpful in guiding appropriate tests and treatments in routine clinical care.

Recent advances in genomic medicine have significantly unveiled the influence of genetic factors in epilepsy. The targeted gene panel approach has been successfully used in specific syndromes and severe epilepsies, such as epileptic encephalopathies and familial epilepsies.(20–27) Hitherto, only two studies have addressed focal epilepsy specifically using targeted gene panel or whole exome sequencing (WES) with targeted gene analysis (28, 29). Here, we developed a more comprehensive focal epilepsy gene panel, with 21 genes, using multiplex polymerase chain reaction (PCR) based technique followed by massively parallel sequencing to study a large cohort of patients with focal epilepsies. We aim to better understand the contribution of currently known disease-causing genes to focal epilepsy and the utility of multi-gene panel in real-world clinical setting.

## Methods

### Patients and phenotyping

Patients with focal epilepsies were recruited for the Department of Neurology, Kaohsiung Chang Gung Memorial Hospital, Taiwan and the Neurology clinic, University of Malaya Medical Center, Malaysia. The clinical information, electroencephalography (EEG) and neuroimaging results were obtained from a direct interview or review of medical records. Most (506/593, 85.3%) of them underwent 3T or 1.5T brain MRI, the remaining 87 had brain CT scans. Patients who had focal structural epilepsy due to stroke, trauma, brain tumor, or focal cortical dysplasia were excluded. Patients with isolated generalized epilepsies were also excluded, but those who have both generalized and focal seizures were still included because some of the genes included in the panel were known to have both presentations. Positive family history was defined as the presence of epilepsy or seizures in the first or second-degree relatives. All of them were recruited regardless of family history and none had received prior genetic testing. All available family members were included for segregation analysis. This study was approved by the local human research ethics committees and written consents were obtained from all subjects. In minors and those with intellectual disabilities, consents were obtained from their legal guardian.

### Focal epilepsy gene panel

Venous blood was obtained and genomic DNA was extracted from peripheral blood leukocytes using QIAGEN DNA extraction kits (Qiagen, Germany), according to the manufacturer instructions (30). A customized focal epilepsy gene panel was used, including 21 genes: *SCN1A, SCN1B, SCN2A, SCN9A, DEPDC5, GRIN2A, GRIN2B, PRRT2, SLC2A1, PCDH19, KCNT1, KCNQ2, KCNQ3, KCNA2, CHRNA4, CHRNB2, CHRNA2, LGI1, GABRG2, HCN1, CHD2*. All coding exons and at least 10 base pair (bp) flanking sequences of the intron/exon boundaries were amplified using targeted specific primers, with a total 69,787 bp region. The amplicon sizes ranged from 204 to 432 bp with an average of 315 bp. Universal primer sequences, 5′-ACACTGACGACATGGTTCTACA-3′ and 5′-TACGGTAGCAGAGACTTGGTCT-3′ were added to the 5' end of all target-specific forward and reverse primers, respectively. Primers were pooled to generate six-plex primer pools per PCR with a final concentration of 1 uM. Libraries were prepared by using the Fluidigm Access 48.48 Array platform (Fluidigm, South San Francisco, California). Harvested amplicon pools underwent another PCR step to barcode the products according to the manufacturer's protocol. Barcoded PCR products were pooled and submitted to an Illumina MiSeq using 2 x 300 bp paired-end runs.

### Bioinformatics analysis

Raw read data was processed with FastQC, FastQ groomer, Trimmomatics to remove primer sequences, and then mapped to human reference genome (version GRCh37) with Burrows-Wheeler Aligner (BWA-MEM, version 0.7.15, http://bio-bwa.sourceforge.net/) (31, 32). The aligned BAM file was processed with SAM tool (http://www.htslib.org/) and Picard (http://picard.sourceforge.net/) to remove low quality mapped reads as well as duplicate reads. Indel realignment was performed using GATK tool as recommended by the Broad Institute GATK Best Practice (33, 34). Single nucleotide variants and small indels were called using FreeBayes (35). The read depth and coverage of each BAM files were calculated using BEDtools (36). Variants that did not adhere to the following criteria were excluded from further analysis: mapping quality <30, base quality <20, coverage <20, variants with strand bias and clustered variants. The variant calling was performed using the Galaxy platform (http://usegalaxy.org). Variants were annotated with wANNOVAR (http://wannovar.wglab.org). Only nonsense, nonsynonymous, splice-site and frameshift variants were further evaluated. Variants presented in the Thousand Genome Project (TGP, http://www.internationalgenome.org/), the Exome Variant Server (EVS, http://evs.gs.washington.edu/EVS/), more than 1 hit in the Board Institute Exome Aggregation Consortium (ExAC, http://exac.broadinstitute.org), and more than five hits in the Genome Aggregation Database (gnomAD, http://gnomad.broadinstitute.org) were excluded (37). Four prediction programs, including SIFT (v1.03) (38), PolyPhen-2 (v2.2.2 build r394) (39), MutationTaster 2 (40), and Combined Annotation Dependent Depletion (CADD v1.2)(41) were used to prioritize variants. The cutoff value of CADD was set at 20. Only variants predicted probably damaging by more than three *in silico* programs were further validated by Sanger sequencing.

### Criteria for pathogenicity of filtered variants

The confirmed rare variants were classified into pathogenic, likely pathogenic, and variants of unknown significance (VUS) modified from previous guidelines (42, 43). Variants presented in the disease databases (HGMD, http://www.hgmd.org/; ClinVar, https://www.ncbi.nlm.nih.gov/clinvar/; LOVD, http://www.lovd.nl/) were classified as being known pathogenic. Null variants (including frameshift mutations, nonsense mutations, obligatory splicing sites mutations, and mutations affecting the initial codon) identified in known epilepsy genes, where loss of function is a known disease mechanism, were also considered to be pathogenic.

Ultra-rare missense variants (not present in TGP, EVS, ≤1 in ExAc and ≤5 in gnomAD) predicted to be deleterious or damaging by more than three of the four prediction programs were classified as likely pathogenic if their phenotypes correlate with the reported literature. If available, functional data and segregation analysis were taken into consideration. Variants that passed *in silico* prediction but the patient's phenotype was not previously associated with the gene were classified as VUS.

### Statistical analysis

Fisher exact test was used for comparison of categorical data. The statistical analysis was performed with R software, version 3.2.1 (44).

## Results

### Patient characteristics

Five hundred and ninety-three patients, including 298 (50.3%) Taiwanese and 295 (49.7%) Malaysian patients, were recruited and underwent customized focal epilepsy gene panel screening. Among them, 315 (53.1%) had temporal lobe epilepsies, 153 (25.8%) frontal lobe epilepsies, 26 (4.4%) occipital lobe epilepsies, 11 parietal lobe epilepsies (1.8%), 13 (2.2%) benign childhood epilepsy with centrotemporal spikes, and 20 (3.4%) had other syndromes with focal seizures, including 12 Dravet syndrome, 5 Lennox-Gastaut syndrome, 2 epilepsy aphasia spectrum disorders and one genetic epilepsy with febrile seizure plus (GEFS+). The localization was undefined in 55 (9.3%) patients. There were 99 (16.7%) patients had a positive family history and the remaining 494 (83.3%) were sporadic cases.

### Customized focal gene panel study

Total 593 patients were screened with the focal epilepsy gene panel with a mean read depth of 142.4x, and 83.8% coverage of the target region for at least 20 reads.

**Figure 1:**
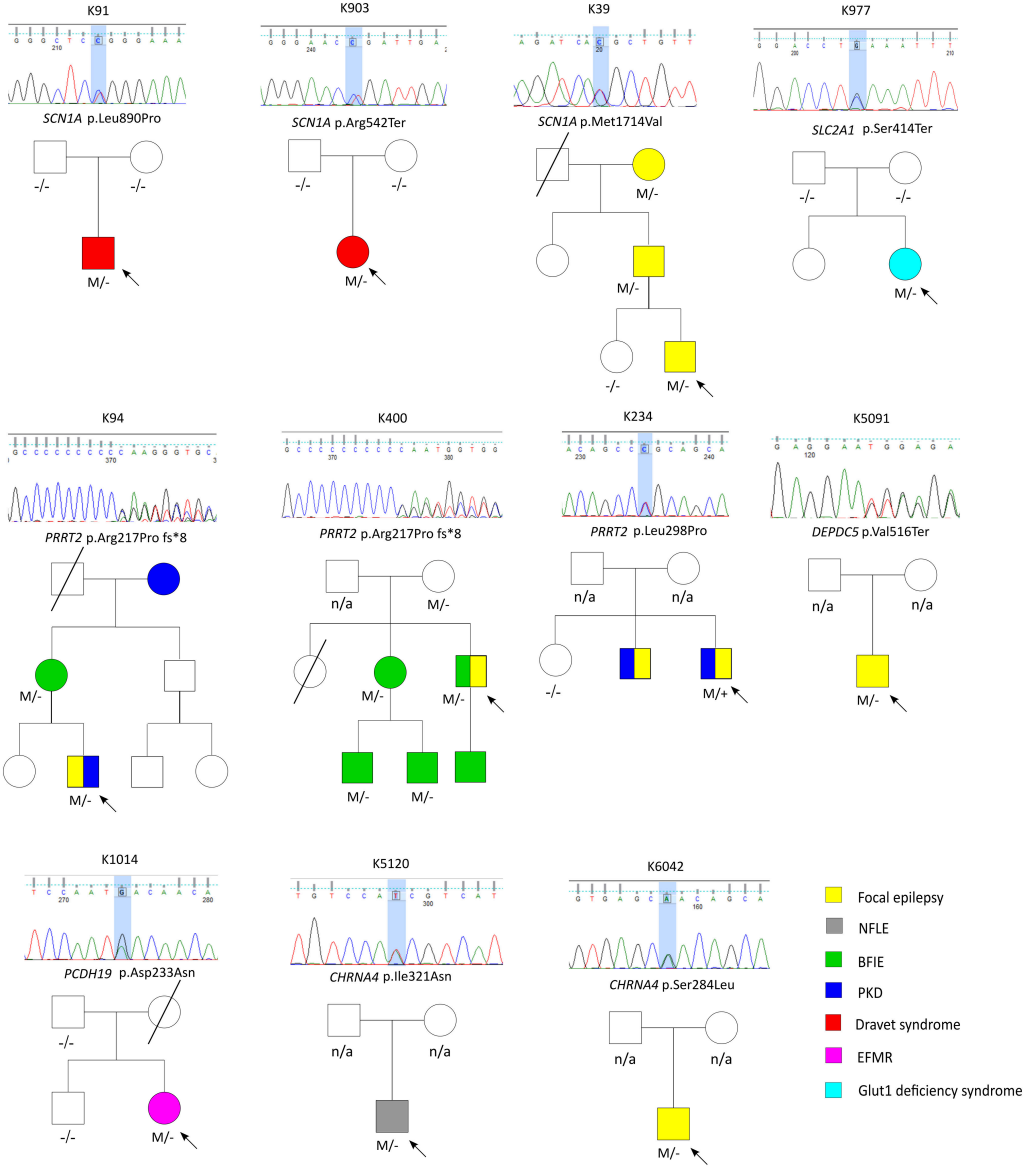
The pedigrees of pathogenic and likely pathogenic variants identified by targeted multigene panel.

A total of 27 variants were confirmed by Sanger sequencing in 25 individuals (4.2%), where two individuals had two different variants. Eleven variants (1.85%) were considered as pathogenic or likely pathogenic, including 4 reported and 7 novel mutations (Table 1); the remaining 16 variants were classified as VUS (Supplemental Table 1). Pathogenic and likely pathogenic variants were found in *SCN1A* (3 patients), *PRRT2* (3 patients), *CHRNA4* (2 patients), followed by one patient each in *DEPDC5, PCDH19*, and *SLC2A1* (Table 1). The pedigrees, clinical phenotypes, and characteristics of patients with pathogenic or likely pathogenic variants were summarized in Figure 1, Table 2 and detailed in below. The clinical phenotypes, and characteristics of patients with variants of unknown significance were summarized in Supplemental Table 2. Pathogenic or likely pathogenic variants were found in 4 out of 99 focal epilepsy patients with a positive family history (4%) compared to 7 out of 494 sporadic focal epilepsy patients (1.4%, *p* = 0.094). We further divided our cohort into patients with specific syndromes and focal epilepsies with intellectual disabilities vs. “non-syndromic” focal epilepsies, the diagnostic rate was higher 12.8% (5/39) in syndromic/ID group than in “non-syndromic” group, which was 1.26% (7/554).

**Table 1:** The pathogenic or likely pathogenic variants identified by customized focal epilepsy gene panel.

**Case**	**Chr**	**Position**	**Ref**	**Alt**	**Genes (RefSeq access number)**	**Type**	**cDNA change**	**AA change**	**Protein Domain**	**gnomAD^†^**	**ExAC^†^**	**SIFT**	**PP2**	**MT**	**CADD**	**Phenotype**	**Inheritance**	**Significance**
K91	2	166894563	A	G	SCN1A (NM_001165963.1)	Missense	c.T2669C	p.Leu890Pro	Transmembrane S5	np	np	D	P	D	26.5	Dravet syndrome	De Novo	Pathogenic
K39	2	166848645	T	C	SCN1A (NM_001165963.1)	Missense	c.A5140G	p.Met1714Val	Intramembrane, pore-forming	np	np	D	P	D	22.6	GEFS+	Inherited	Likely pathogenic
K903	2	166848879	G	A	SCN1A (NM_001165963.1)	Stopgain	c.C4906T	p.Arg1636Ter	Transmembrane	np	np	n/a	n/a	D	43	Dravet syndrome	De Novo	Pathogenic
K94	16	29825024	-	C	PRRT2 (NM_145239.2)	Frameshift	c.649dupC	p.Arg217Profs^*^8	Extracellular	np	np	n/a	n/a	n/a	n/a	Focal epilepsy	Inherited	Pathogenic
K400	16	29825024	-	C	PRRT2 (NM_145239.2)	Frameshift	c.649dupC	p.Arg217Profs^*^8	Extracellular	np	np	n/a	n/a	n/a	n/a	Infantile epilepsy and late focal epilepsy	Inherited	Pathogenic
K234	16	29825667	T	C	PRRT2 (NM_145239.2)	Missense	c.T893C	p.Leu298Pro	Cytoplasmic	np	np	D	D	D	26.1	Focal epilepsy and PKD	n/a	Pathogenic
K6042	20	61981912	G	A	CHRNA4 (NM_000744.6)	Missense	c.C851T	p.Ser284Leu	Transmembrane	np	np	D	D	A	32	Nocturnal focal epilepsy	n/a	Pathogenic
K5120	20	61981801	A	T	CHRNA4 (NM_000744.6)	Missense	c.T962A	p.Ile321Asn	Transmembrane	np	np	D	D	D	27.9	NFLE	n/a	Likely pathogenic
K5091	22	32211078	G	-	DEPDC5 (NM_001242896.1)	Stopgain	c.1546delG	p.Val516Ter	No information	np	np	n/a	n/a	n/a	n/a	Focal epilepsy	n/a	Pathogenic
K1014	X	99662899	C	T	PCDH19 (NM_00118488.0)	Missense	c.G697A	p.Asp233Asn	Extracellular, Calcium binding pocket	np	np	D	D	D	28.2	EFMR	De Novo	Pathogenic
K977	1	43393313	G	C	SLC2A1 (NM_006516)	Stopgain	c.C1241G	p.Ser414Ter	Transmembrane	np	np	D	n/a	D	42	Glut1 deficiency syndrome	De Novo	Pathogenic

Chr, Chromosome; AA, Amino Acid; gnomAD, Genome Aggregation Database; TPG, Thousand Genome Project; ExAc, Exome Aggregation Consortium; EVS, Exome Variant Server; PP2, PolyPhen2; MT, Mutation Taster; CADD, Combined Annotation Dependent Depletion ; n/a, not available; np, not present; NFLE, nocturnal frontal lobe epilepsy; GEFS+, genetic epilepsy and febrile seizure plus; FS, febrile seizures; EFMR, epilepsy and mental retardation limited to female; PKD, paroxysmal kinesigenic dyskinesia.

† *All variants were not present in the TGP or EVS databases*.

**Table 2:** The clinical phenotypes and characteristics of patients presented with pathogenic or likely pathogenic variants.

**Case**	**Age/Gender**	**Gene**	**Diagnosis**	**Onset**	**Seizure type**	**EEG**	**Neuroimaging**	**Frequency**	**FH**
K91	26/M	SCN1A	Dravet syndrome	8 months	Fever-related alternative hemi-clonic focal seizures, BTCS	Left temporal focal spikes	Normal	3–5/month	No
K39	55/M	SCN1A	GEFS+	3	Generalized seizures, occasionally focal seizures	Normal	Normal	1–2/year	Yes, his son has FS 3 months old and recurrent seizure at age of 15, both focal and generalized epilepsies, mother had elderly onset seizures after stroke
K903	19/F	SCN1A	Dravet syndrome	2 month for FS, 2.5 for afebrile seizure	Staring, BTCS, focal, and myoclonic jerks	Multifocal epileptiform discharges	Diffuse brain atrophy	1/week	No
K94	36/F	PRRT2	Focal epilepsy	7	Strange sensation –> BTCS	Normal	Normal	No seizure for years	Yes, son has infantile seizure 4 month
K400	31/M	PRRT2	BFIE, late focal epilepsy	4 months, recurrent at 18	BTCS	Right focal spike over central area	Right hippocampal and right anterior temporal arachnoid cyst	No seizure for years	Yes
K234	24/M	PRRT2	Focal epilepsy and PKD	21	Visual symptoms, ictal cry, BTCS	Focal left T-O sharp waves	Normal	No seizure for 2 years	Yes, brother has PKD
K6042	38/M	CHRNA4	Nocturnal focal epilepsy	7	Nocturnal BTCS	Right temporal sharp waves	Norma	Seizure free 1 year	No
K5120	44/M	CHRNA4	NFLE	16	Wandering at night with irrelevant verbal response	Right temporal theta activities	Normal	Seizure free 6 months	unknown
K5091	45/M	DEPDC5	Focal epilepsy	23	Dizziness then BTCS	Right hemisphere slow	Normal	1/year	No
K1014	33/F	PCDH19	EFMR	9 month	Staring episode, myoclonic, BTCS	Bilateral temporal epileptiform discharges	Normal	4–6 seizures/month	No
K977	8/F	SLC2A1	Glut1 deficiency syndrome	8 months	Focal seizure with impaired consciousness	Right frontal epileptiform discharges	Delayed myelination over periventricular area	1–2/year on ketogenic diet	No

*FICS, focal impaired awareness seizures; BTCS, bilateral tonic-clonic seizures; EEG, electroencephalography; T, temporal; O, occipital; NFLE, nocturnal frontal lobe epilepsy; GEFS+, genetic epilepsy and febrile seizure plus; FS, febrile seizures; EFMR, epilepsy and mental retardation limited to female; FH, family history*.

### SCN1A

Three variant were found in *SCN1A*, including two patients with Dravet syndromes (p.Leu890Pro, p.Arg1636Ter) and one family with genetic epilepsy with febrile seizure plus (GEFS+) (p.Met1714Val, Figure 1). The missense mutation p.Leu890Pro is *de novo* and located in the pore-forming transmembrane S5 domain, while the inherited missense mutation in GEFS+ family (p.Met1714Val) is located in the pore-forming loop between S5 and S6 domain. Both are novel mutations and located in the hot-spot for disease-related missense mutations (45). The p.Met1714Val missense variant was also found in the affected son and proband's mother who had focal seizures in old age (Figure 1).

The patient with de novo p.Leu890Pro mutation had more than 10 seizures a month on Carbamazepine, Vigabatrin and Levetiracetam before the genetic diagnosis. His medication was changed to Topiramate, Levetiracetam and Clobazam in the following months after receiving the results and his seizure frequency drastically reduced to only 1-2 seizure a month.

### PRRT2

Three variants were found in *PRRT2* gene, including two hotspot p.Arg217Profs^*^8 frameshift mutations that were inherited in the families with benign infantile epilepsies (Figure 1). The third missense variant p.Leu298Pro is novel and found in a patient with both focal epilepsy and paroxysmal kinesigenic dyskinesia. *In silico* programs predicted this missense mutation to be deleterious/damaging/disease causing. The available unaffected sister did not have the mutation, the affected brother had paroxysmal kinesigenic dyskinesia and epilepsy but was not available for testing. Functional study of this variant showed lack of membrane localization of the mutant protein, similar to the hotspot truncating mutation p.Arg217Profs^*^8 (Tsai et al., under review). Therefore, the variant is classified as pathogenic based on consistent phenotype and functional data.

### CHRNA4

Two missense variants (p.Ser284Leu, p.Ile321Asn) were found in *CHRNA4*, both located in the transmembrane domain; the novel missense variant p.Ile321Asn is predicted to be disease-causing by all *in silico* programs. Both patients had nocturnal frontal lobe epilepsy.

### DEPDC5

The pathogenic nonsense mutation p.Val516Ter in *DEPDC5* is not presented in any control databases and is predicted to cause nonsense-mediated decay. Therefore, the mutation is likely to cause haploinsufficiency of the DEPDC5 protein, consistent with the currently known molecular mechanism. Clinically, the patient had focal epilepsy, consistent with the *DEPDC5* phenotypic spectrum.

### PCDH19

The patient had febrile seizures at 9 months old and later developed fever sensitive seizure clusters and intellectual disability. The novel missense variant p.Asp233Asn is located in the extracellular domain; the amino acid forms part of the calcium binding pocket that is critical to the homophilic binding function of PCDH19. The variant is classified as de novo pathogenic because both unaffected parents did not carry the mutation.

### SLC2A1

The patient had early onset focal seizures, intellectual disability, and low CSF glucose level, and clinically suspected GLUT1 deficiency syndrome. The novel nonsense variant p.Ser414Ter is located in the transmembrane domain and both unaffected parents did not have the mutation, thus the variant is classified as de novo pathogenic. The patient received ketogenic diet and responded partially to the therapy.

## Discussion

The real-world utility and experience of the multi-gene panel in focal epilepsies, the most common form of epilepsies, is very limited (28, 29). We screened a large cohort of focal epilepsy patients and found that 1.85% (11/594) can be attributed to a pathogenic or likely pathogenic variant. Our study highlights the usefulness but also challenges and limitations of using the multi-gene panel in focal epilepsies. The determination of the significance of identified genetic variants is complicated in real-world situation, which requires correct correlation between phenotypes and genotypes. It becomes more difficult when the phenotypes are not previously associated with the genes where variants are identified. It requires more studies to explore the boundaries of the phenotypic spectrum associated with each epilepsy gene. Some of the VUS may be reclassified as pathogenic or likely pathogenic when the phenotype-genotype relationship redefined. Moreover, we noted lack of segregation data is a common obstacle due to limited availability of the family members in routine clinical setting, which makes the determination of the pathogenicity of variants more difficult.

Previous studies using multi-gene panel in epilepsy with various genes (*n* = 35–327) have generated a diagnostic yield ranged from 10 to 48.5% (11, 21, 23–27, 32). Those studies selected patients with epileptic encephalopathy (21, 27), epileptic syndrome with suspected genetic etiology (12, 21, 32) or enriched for positive family history (21). The higher diagnostic yield was likely due to early onset epilepsies and severe/specific phenotypes such as epileptic encephalopathies (21), which are known to have a stronger genetic underpinning.

Our results are consistent with those reported by Hildebrand et. al., suggesting that currently known focal epilepsy genes only explain a small proportion (0.8–1.85%) of all focal epilepsy patients (29). After excluding patients with clinical suspected specific epilepsy syndromes, such as Dravet syndrome, GEFS+, EFMR and patients with intellectual disability, the diagnostic rate for “garden-variety” focal epilepsies was 1.26%. The reason for the slightly higher diagnostic rate in our study could be explained by the fact that most of our patients had not previously received genetic testing and 10 more genes were included in our panel (29). Interestingly, a recent study used WES based targeted gene analysis of 64 genes on 40 consecutive patients with focal epilepsies with suspected genetic etiology. (28) They reported a much higher positive rate at 12.5% (5/40), three variants were found when limiting to the 21 genes we studied (3/40, 7.5%). The higher yield rate could be explained by the presence of a positive family history of this study. In our study, patients with a positive family history also have a higher diagnostic rate (4% vs. 1.4%) although not statistically significant. Moreover, we did not include copy number variation (CNV), in-frame indels and splice-region variants that are not on the canonical site in this study, which may underestimate the diagnostic rate.

Taken together, our study found a multi-gene panel provides genetic diagnosis of a relatively small percentage of real-world patients with focal epilepsies. Our data indicate that the underlying genetic architecture of focal epilepsies is very heterogeneous and more genes await discovery. Supporting this, a recent study using WES reported positive findings in 38% of patients with focal epilepsy, including discovery of novel genes in 7% (46). The positive rate is expected to increase in the future when more causative genes are identified in focal epilepsy. Obtaining a correct genetic diagnosis is important as it may alter the clinical decision on epilepsy surgery, selection of antiepileptic drugs and reproductive counseling (28). In routine clinical care, careful selection of patients with specific phenotypes/syndromes or positive family histories, adopting a broader panel with more genes, or using WES or even whole genome sequencing are likely to further increase the diagnostic yield.

## Ethic approval and consents to participate

This study was approved by the local human research ethics committees (Chang Gung Medical Foundation Institutional Reviewer Board 104-2308B and University Malaya Medical Centre Medical Research Ethics Committee 944.3) and written consents were obtained from all subjects.

## Author contributions

C-KC, Y-CC, C-HL, C-WL, W-NC, C-CN, and K-SL contributed to the acquisition and interpretation of the data and revising the manuscript for intellectual content. M-HT and D-YH contributed to the design and conceptualization of the study; analysis and interpretation of the data; drafting, revising and final approval of the manuscript for intellectual content.

### Conflict of interest statement

The authors declare that the research was conducted in the absence of any commercial or financial relationships that could be construed as a potential conflict of interest. The handling Editor declared a past coauthorship with one of the authors M-HT.
