# Supplementary material for: Molecular Genetic Characterization of Patients With Focal Epilepsy Using a Customized Targeted Resequencing Gene Panel
Source: Front Neurol. 2018 Jul 6;9:515. doi: 10.3389/fneur.2018.00515 (PMC6043663; doi:10.3389/fneur.2018.00515)
Supplement: Supplementary file 1 [file Data_Sheet_1.docx]

**Supplement Table 1 The rare variants with unknown significance identified by customized focal epilepsy gene panel**

| Case | Chr | Position | Ref | Alt | Genes | Type | cDNA change | AA change | Protein Domain | gnomAD† | ExAC† | SIFT | PP2 | MT | CADD | Phenotype | Inheritance | Significance |
| --- | --- | --- | --- | --- | --- | --- | --- | --- | --- | --- | --- | --- | --- | --- | --- | --- | --- | --- |
| K416 | 20 | 61981831 | A | C | CHRNA4 | Missense | c.T932G | p.Leu311Arg | Transmembrane | np | np | D | D | D | 25.7 | Focal epilepsy | n/a | VUS |
| K5402 | 22 | 32272245 | G | A | DEPDC5 | Missense | c.G3799A | p.Asp1267Asn | No information | 1 | np | D | B | D | 26.2 | TLE | n/a | VUS |
| K194 | 9 | 138594134 | G | - | KCNT1 | Frameshift | c.30delG | p.Gly12Alafs*43 | Cytoplasmic | np | np | n/a | n/a | n/a | n/a | Focal epilepsy and psychosis | n/a | VUS |
| K488 | 9 | 138661850 | G | T | KCNT1 | Missense | c.G1433T | p.Cys478Phe | Cytoplasmic | np | np | D | D | D | 28.9 | Focal epilepsy | n/a | VUS |
| K79 | 9 | 138661885 | C | T | KCNT1 | Missense | c.C1468T | p.His490Tyr | Cytoplasmic | np | np | D | D | D | 27.9 | Occipital lobe epilepsy | n/a | VUS |
| K5495 | 8 | 27321175 | C | T | CHRNA2 | Missense | c.G785A | p.Arg262Gln | Transmembrane | np | np | D | D | D | 32 | Left posterior TLE | n/a | VUS |
| K107 | 8 | 27321250 | G | A | CHRNA2 | Missense | c.C710T | p.Thr237Met | Extracellular | np | np | D | P | D | 26.7 | Focal epilepsy with intellectual disability | n/a | VUS |
| K161 | 1 | 154548252 | G | C | CHRNB2 | Missense | c.G1353C | p.Trp451Cys | Transmembrane | 2 | 1 | D | D | D | 33 | Focal epilepsy | n/a | VUS |
| K14 | 16 | 10032327 | C | T | GRIN2A | Missense | c.G496A | p.Asp166Asn | Extracellular | 1 | np | D | D | D | 25.1 | Focal epilepsy | n/a | VUS |
| K14 | 12 | 13717464 | C | T | GRIN2B | Missense | c.G2708A | p.Arg903His | Cytoplasmic | np | np | D | D | D | 31 | Focal epilepsy | n/a | VUS |
| K207 | 8 | 133144501 | A | T | KCNQ3 | Missense | c.T1810A | p.Tyr604Asn | Cytoplsamic | np | np | T | D | D | 24.7 | NFLE | n/a | VUS |
| K5150 | 8 | 133150198 | T | C | KCNQ3 | Missense | c.A1634G | p.Asp545Gly | Cytoplsamic | np | np | D | D | D | 31 | Occipital lobe epilepsy | n/a | VUS |
| K5182 | 8 | 133150214 | G | A | KCNQ3 | Missense | c.C1618T | p.Pro540Ser | Cytoplsamic | np | np | D | D | D | 31 | FS, TLE+HS | n/a | VUS |
| K5115 | 8 | 133182633 | G | T | KCNQ3 | Missense | c.C1183A | p.Leu395Met | Cytoplsamic | np | np | D | D | D | 26.8 | TLE | n/a | VUS |
| K518 | 8 | 133459465 | A | - | KCNQ3 | Frameshift | c.9delT | p.Ala4Glnfs*8‡ | Cytoplsamic | np | np | n/a | n/a | n/a | n/a | Bi-temporal lobe epilepsy | n/a | VUS |
| K5182 | 2 | 167141324 | C | T | SCN9A | Missense | c.G1613A | p.Ser538Asn | Cytoplasmic | np | np | D | D | D | 22.8 | TLE+HS | n/a | VUS |

Abbreviations: Chr, Chromosome; AA, Amino Acid; gnomAD, Genome Aggregation Database ; TPG, Thousand Genome Project; ExAc, Exome Aggregation Consortium; EVS, Exome Variant Server; PP2, PolyPhen2; MT, Mutation Taster; CADD, Combined Annotation Dependent Depletion ; n/a, not available; np, not present; VUS, variant of unknown significance; TLE, temporal lobe epilepsy; HS, hippocampal sclerosis; NFLE, nocturnal frontal lobe epilepsy; GEFS+, genetic epilepsy and febrile seizure plus; FS, febrile seizures; EFMR, epilepsy and mental retardation limited to female; PKD, paroxysmal kinesigenic dyskinesia

† All variants were not present in the TGP or EVS databases

‡ On alternate exon1 of isoform 2 transcript: NM_001204824

**Supplemental Table 2 The clinical phenotypes and characteristics of patients presented with variants of unknown significance**

| **Case** | **Age/Gender** | **Gene** | **Diagnosis** | **Onset** | **Seizure type** | **EEG** | **Neuroimaging** | **Frequency** | **FH** |
| --- | --- | --- | --- | --- | --- | --- | --- | --- | --- |
| K5495 | 39/M | CHRNA2 | Left posterior TLE | 12 | Visual aura, FICS with automatism | Left posterior temporal sharp waves | Left temporal atrophy | 4-5/year | No |
| K107 | 46/F | CHRNA2 | Focal epilepsy with intellectual disability | Childhood | FICS, BTCS | Not available | Bilateral frontal atrophic change | 2-3/week | No |
| K416 | 46/F | CHRNA4 | Focal epilepsy | FS at 9 mo and recurrent at age of 16 | Dizziness, both hand numbness --> BTCS with head deviation to left | Focal right parietal slowing | Normal | Seizure free | No |
| K161 | 30/M | CHRNB2 | Focal epilepsy | 25 | Flashlight then BTCS | Normal | Normal | No seizure for 2 years | Yes |
| K5402 | 39/F | DEPDC5 | TLE | 29 | déjà vu and oral automatism, FICS | Left temporal epileptiform discharges | Normal | 2/month | No |
| K14 | 59/F | GRIN2A, GRIN2B | Nocturnal focal epilepsy | Childhood | FICS | No epileptiform discharges | Normal | No seizure | No |
| K207 | 42/F | KCNQ3 | NFLE |  | FICS, BTCS | Bilateral frontal epileptiform discharge | Normal | 1/3month | No |
| K5150 | 68/F | KCNQ3 | Occipital lobe epilepsy | 30 | Visual aura and BTCS | Normal | Normal | Seizure free 5 years | No |
| K5182 | 46/M | KCNQ3, SCN9A | FS, TLE+HS | 2 for FS, 40 for TLE | Blank staring with limb automatism | Bilateral (L>R) temporal interictal epileptiform discharges | Left HS | 1/month | No |
| K5115 | 63/M | KCNQ3 | Temporal lobe epilepsy | 13 | Blank staring and BTCS | Right temporal epileptiform discharges | Normal | Seizure free for 2 years | unknown |
| K518 | 47/M | KCNQ3 | Bi-temporal lobe epilepsy | 45 | Mouth angle twitching, FICS --> BTCS | Right and left temporal epileptiform discharges | Limbic area hyper-signal change | 3-4/month | No |
| K194 | 46/M | KCNT1 | Focal epilepsy and psychosis | 31 | FICS, BTCS | Focal right T-O slowing | Normal | 1/2-3 years | No |
| K488 | 35/M | KCNT1 | Focal epilepsy | 18 | FICS, BTCS | No epileptiform discharges | Normal | 1/2 years | Yes, distant relatives |
| K79 | 50/F | KCNT1 | Occipital lobe epilepsy | 14 | Visual flashlights | Right temporal spikes | Right hemiatrophy | No seizure for years | No |

FICS, focal impaired awareness seizures; BTCS, bilateral tonic-clonic seizures; EEG, electroencephalography; T, temporal; O, occipital; TLE, temporal lobe epilepsy; HS, hippocampal sclerosis; NFLE, nocturnal frontal lobe epilepsy; FS, febrile seizures; FH, family history
